# Effects of Hyperedge Overlap and Internal Structure on Hypernetwork Synchronization Dynamics

**DOI:** 10.3390/e27090889

**Published:** 2025-08-22

**Authors:** Hong-Yu Chen, Xiu-Juan Ma, Fu-Xiang Ma, Hai-Bing Xiao

**Affiliations:** 1 School of Computer, Qinghai Normal University, Xining 810008, China; 202333331030@stu.qhnu.edu.cn (H.-Y.C.);; 2 The State Key Laboratory of Tibetan Intelligence, Xining 810008, China

**Keywords:** hypernetwork, hyperedge overlap, hyperedge internal structure, projected graph, synchronization

## Abstract

The internal structure of hyperedges has become central to understanding collective dynamics in hypernetworks. This study investigates the impact of hyperedge overlap on network synchronization when hyperedge structures are explicitly considered. We propose a modified hyper-adjacency matrix that captures the internal organization of the hyperedges while preserving the higher-order properties. Using this framework, we examine how non-complete connections within hyperedges influence synchronization as the overlap increases. Our findings reveal clear differences from fully connected hyperedge models. Furthermore, spectral graph theory and numerical simulations confirm that the structural variations induced by overlaps significantly regulate global synchronization. This work extends the theoretical framework of hypernetwork synchronization and highlights the critical role of hyperedge overlaps in shaping the internal hyperedge structure.

## 1. Introduction

High-order networks have emerged as a powerful modeling framework for characterizing multiple interactions in complex systems [1,2,3,4,5]. Compared with traditional complex networks, high-order networks can more precisely capture intricate group interactions and collective association patterns, rather than merely representing pairwise relationships. Owing to their notable advantages in describing multiple interactions, high-order networks have experienced substantial development and widespread application over the past decade [6,7,8]. Two common forms of high-order networks include simplicial complexes and hypernetworks. Simplicial complexes are characterized by considering all of the subfaces of the complex [9]. In contrast, a hyperedge does not imply the existence of any subsets as hyperedges in the same hypergraph [10]. In response to the diverse properties of high-order networks and the modeling requirements of complex systems, extensive theoretical and applied studies on the modeling and dynamical processes of various types of high-order networks have been conducted in recent years [11,12,13].

**Figure 1:**
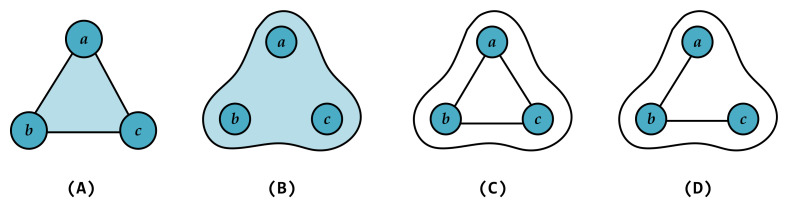
A comparison between simplex and hyperedge structures. In each high-order structure, there are three nodes {a,b,c}. Panel (**A**) shows a 2-simplex. Panel (**B**) shows a three-node hyperedge. Panels (**C**,**D**) show two different connection patterns within the hyperedge in (**B**).

In the study of complex systems, researchers often abstract systems into high-order networks to characterize their higher-order interactions. A common assumption in such models is that nodes within a high-order structure are fully connected. However, many real-world complex systems do not necessarily conform to this idealized assumption. Similar to simplicial complexes, hypernetworks allow a hyperedge to simultaneously associate multiple nodes. Yet in simplicial complexes, all of the subfaces of the complex must exist by definition. For example, using a simplicial complex to represent the relationship between three nodes {a,b,c} assumes that the subfaces {a,b}, {a,c}, and {b,c} all exist, along with the individual nodes. In contrast, hypernetworks do not require all connections between nodes within a hyperedge to be present [14]. Specifically, in the higher-order structure shown in Figure 1, subfigure (A) shows a 2-simplex, which includes one face, three edges, and three nodes. Subfigure (B) shows a hyperedge with three nodes. Subfigures (C) and (D) show two possible connection patterns within the hyperedge in (B): (C) is fully connected, while (D) is not fully connected. This feature enables hypernetworks to more accurately reflect the diverse interaction patterns observed in real-world systems. For example, within a group chat, all members belong to the same chat group, yet some individuals may never have directly communicated and may not even know each other. Similarly, in ecological systems, although multiple species may coexist within the same environment, there may be no direct biological interactions among them. In such cases, hypernetworks are used as a more flexible model that accommodates locally incomplete connections. In other words, the nodes within a hyperedge in a hypernetwork are not necessarily fully connected. This modeling approach not only captures multi-node higher-order relationships but also retains the internal complexity of the system, thereby demonstrating enhanced applicability and flexibility. Currently, studies on the properties of hypernetworks that consider the internal structure of the hyperedges appear only in the research on robustness. Zhou et al. [15] studied how the robustness of hypernetworks changes with different internal connection patterns for the hyperedges, based on the capacity-load model. However, in the study of synchronization dynamics, research considering the internal structure of the hyperedges is still lacking.

Synchronization, as a widely observed natural phenomenon, plays a significant role in the study of complex system dynamics. In recent years, synchronization on hypernetworks has attracted considerable attention. Sorrentino et al. [16] investigated the synchronization problem for coupled dynamical systems in hypernetworks, considering the coexistence of different types of interactions within the same system. Krawiecki et al. [17] studied chaotic synchronization on complex hypergraphs, focusing on chaotic oscillators placed on the nodes and nonlinear couplings defined via hyperedges connecting groups of oscillators. Adhikari et al. [18] used a hypergraph generative model and developed a mean-field analysis using dimensionality reduction techniques to obtain low-dimensional descriptions of synchronization in terms of the hypergraph’s structural parameters. These studies have primarily approached hypernetwork synchronization from a global perspective, constructing synchronization models and analyzing their influencing factors. Beyond global properties, researchers have also investigated local structural features that affect synchronizability in hypernetworks. One key feature is hyperedge overlap, referring to the phenomenon where multiple hyperedges share common nodes [19]. Hyperedge overlap significantly contributes to the structural complexity of hypernetworks and has substantial effects on their synchronization dynamics [20]. In recent years, research on the synchronization in hypernetworks with hyperedge overlaps has deepened. Malizia et al. [20] further revealed that hyperedge overlaps not only influence the synchronization ability but can also trigger explosive transitions. Santiago et al. [21] demonstrated that the degree of hyperedge overlap plays a critical role in determining the synchronizability in systems with higher-order interactions. Notably, in the aforementioned studies, hyperedges have typically been assumed to represent fully connected high-order relationships. The potential heterogeneity within hyperedges is often neglected. At the same time, although their methods for describing the hypernetwork structure effectively capture the higher-order structure of the hyperedges and their relationships, they cannot represent the internal structure within the hyperedges. Therefore, there remains a lack of research addressing the relationship between hyperedges’ internal structures and synchronization performance. Whether these internal connection patterns influence global dynamical behavior is an open-ended question.

At the same time, in ordinary networks where only nodes interact in pairs, researchers have found an influence of the network structure on synchronization ability. In network structures, the number and distribution of the edges directly affect the interaction strength between nodes and the overall coordination capability [22]. Generally, the more edges there are, the stronger the coupling strength of the network. This allows information, energy, or state variables to be transmitted more efficiently among nodes. Therefore, the synchronizability of the network will be significantly enhanced [23,24]. Especially in undirected networks, an increase in the number of edges leads to a wider gap between the eigenvalues of the Laplacian matrix, with the second smallest eigenvalue typically increasing accordingly [25]. This implies that the network is more likely to achieve synchronization. Another important structural feature in networks is the cycle. A cycle in a network is simply defined as a closed (non-repeating) path with the same starting and ending node [26]. Cycle structures are key components of networks and promote network functions according to many aspects. The node centrality defined by cycle structures performs well for diffusion and control processes [27]. Networks with cycle structures exhibit optimal synchronizability [28]. Based on these conclusions, we investigate whether hyperedge overlap leads to changes in such substructures when considering hyperedges’ internal structures. Therefore, we explored whether hyperedge overlap, when considering the internal structure of the hyperedges, could lead to changes in certain substructures, which might further affect the global synchronizability of the hypernetwork.

In this paper, we propose a new method for constructing a high-dimensional matrix. This method creates a matrix different from the hyper-adjacency matrix, which we call the modified hyper-adjacency matrix, to describe the connections between nodes within a hyperedge. In this way, the modified hyper-adjacency matrix characterizes the connectivity pattern inside the hyperedge without neglecting the high-order properties of the hyperedge. By employing an equivalent dimensionality reduction approach alongside the calculation of the node degrees in each hyperedge, we construct a Laplacian matrix that incorporates the internal structures of the hyperedges to analyze the synchronizability of hypernetworks. Furthermore, based on this framework, we investigate how hyperedge overlap affects the synchronization dynamics when the hyperedges’ internal structures are taken into account. In hypernetworks containing hyperedges with randomly connected internal structures, we theoretically demonstrate that increasing the hyperedge overlap promotes higher connection probabilities among the nodes within the hyperedges. To characterize the substructures within the hyperedges further, we utilize metrics such as the average degree, global clustering coefficient, and average closeness in the projected graph to quantify the influence of the hyperedge overlap on the formation of hypernetwork substructures. Our findings reveal that a greater hyperedge overlap facilitates the emergence of richer substructures within the hyperedges, which in turn enhances the global synchronizability of the hypernetwork. Finally, using empirical networks with identical node sets but differing relational structures, we perform an eigenvalue analysis and numerical simulations to validate the impact of hyperedge overlap on the synchronization performance in hypernetworks further, with the hyperedges’ internal structures explicitly considered.

The rest of this paper is organized as follows. Section 2 introduces the hyper-adjacency matrix with internal structure corrections for the hyperedges, the hypernetwork synchronization model, and its Laplacian matrix. Section 3 shows the impact of hyperedge overlap on the internal structure of the hyperedges. Section 4 verifies our conclusions further in an empirical network. Section 5 concludes this paper.

## 2. Model and Methods

### 2.1. The Hypernetwork and the Hyper-Adjacency Matrix

**Figure 2:**
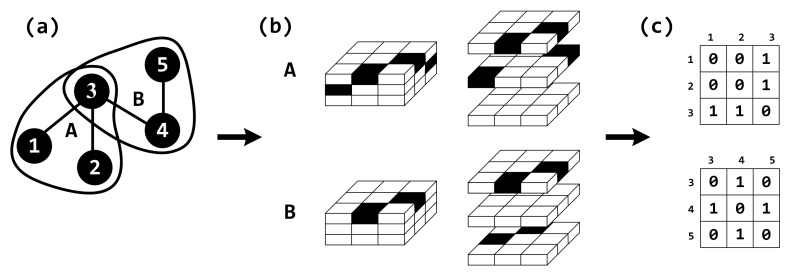
A schematic diagram of dimensionality reduction for hypernetworks. Panel (**a**) shows a uniform hypernetwork with 5 nodes and 2 hyperedges. Among them, the nodes are 1–5, and the hyperedges are A and B. Panel (**b**) depicts a hyper-adjacent matrix with internal structure correction for the hyperedges in (**a**). Panel (**c**) represents the result of hyper-adjacent matrix dimensionality reduction in (**b**).

Consider a hypergraph H=(V,E) consisting of a set $V=\{v_{1},v_{2},\ldots ,v_{N}\}$ of *N* nodes and a set $E=\{e^{\left(1\right)},e^{\left(2\right)},\ldots ,e^{\left(M\right)}\}$ of *M* hyperedges, where $e^{\left(l\right)}\neq \emptyset$ for l=1,2,…,M [29]. Let $k^{\left(l\right)}$ denote the number of nodes contained in the hyperedge $e^{\left(l\right)}$, which is also referred to as the size of the hyperedge in this paper. If every hyperedge in the hypergraph *H* contains exactly *k* nodes, i.e., $k^{\left(l\right)}=k$, then *H* is referred to as a uniform hypergraph. Conversely, if at least two hyperedges exist in *H* with different numbers of nodes, *H* is termed a non-uniform hypergraph. Hyperedge overlap focuses on the number of nodes in common between hyperedges [21]. We use the function $o\left(e_{i},e_{j}\right)=\left|e_{i}\cap e_{j}\right|$ to measure the overlap between hyperedges $e_{i}$ and $e_{j}$. Here, $o(e_{i},e_{j})$ denotes the number of nodes shared by the two hyperedges. Figure 2a shows a uniform hypergraph with 5 nodes and 2 hyperedges. A network based on a hypergraph is called a hypernetwork.

For hypernetworks, since the interactions between the nodes in each hyperedge are through high-order relationships rather than just pairwise interactions, we use the hyper-adjacency matrix to describe the relationships between nodes in the hyperedge. The hyper-acas

(1) $$A_{H_{k}}={\left(a_{v_{i_{1}},v_{i_{2}},\cdots ,v_{i_{k}}}\right)}_{\underset{k}{\underset{︸}{N\;\times \;N\;\times \;\cdots \;\times \;N}}},\;\left(v_{i_{1}},v_{i_{2}},\cdots ,v_{i_{k}}\in \left\{1,2,\cdots ,N\right\}\right),$$

where $A_{H_{k}}$ is a *k*-dimensional, *N*-order symmetric matrix. The element $a_{v_{i_{1}},v_{i_{2}},\ldots ,v_{i_{k}}}$ represents the adjacency relationship among any *k* nodes in the hypergraph, defined as

(2) $$a_{v_{i_{1}},v_{i_{2}},\cdots ,v_{i_{k}}}=\left\{\begin{array}{cc} 1, & \left\{v_{i_{1}},v_{i_{2}},\cdots ,v_{i_{k}}\right\}\subseteq \exists e_{i},\;i=1,2,\cdots ,M\\ 0, & \left\{v_{i_{1}},v_{i_{2}},\cdots ,v_{i_{k}}\right\}⊈\forall e_{i},\;i=1,2,\cdots ,M \end{array}\right.,$$

Here, $A_{H_{k}}$ is referred to as the hyper-adjacency matrix of the hyperedge [30].

In conventional hyper-adjacency matrix formulations, the internal structure of a hyperedge is typically described by assuming full connectivity among its constituent nodes, in order to represent higher-order relationships. For example, if nodes *i* and *j* belong to a two-node hyperedge, the corresponding entry $a_{ij}=1$; otherwise, $a_{ij}=0$. Similarly, for a three-node hyperedge, $a_{ijk}=1$ if and only if nodes *i*, *j*, and *k* are all included in the same hyperedge. All combinations of *i*, *j*, and *k* must be 1, which means that there is an edge between any combinations of these three nodes.

The existing methods for constructing hyper-adjacency matrices assume that the nodes within a hyperedge are fully connected. However, when the internal structure of the hyperedges is considered in more detail, traditional hyper-adjacency matrices often overrepresent the connections. To address this, we propose a new construction method based on the existing hyper-adjacency matrix which can accurately capture the internal connectivity of the hyperedges without adding redundant or missing information. Taking a hyperedge containing nodes *i*, *j*, and *k* as an example in Figure 2, if there is no direct interaction between nodes *i* and *j*, then based on the conventional hyper-adjacency matrix, we set $a_{kij}=a_{kji}=0$. This is because while the higher-order interaction among nodes *i*, *j*, and *k* exists, the interaction between nodes *i* and *j* is absent. More generally, in the hyper-adjacency entry $a_{v_{i_{1}},v_{i_{2}},\ldots ,v_{i_{k}}}$, if there is no direct interaction between a pair of nodes $\left(v_{i_{p}},v_{i_{q}}\right)$ with p≠q, then

(3) $$\begin{array}{cc} \; & a_{v_{i_{1}},v_{i_{2}},\cdots ,v_{i_{p-1}},v_{i_{p+1}},\cdots ,v_{i_{q-1}},v_{i_{q+1}},\cdots ,v_{i_{k}},v_{i_{p}},v_{i_{q}}}\\ \; & =a_{v_{i_{1}},v_{i_{2}},\cdots ,v_{i_{p-1}},v_{i_{p+1}},\cdots ,v_{i_{q-1}},v_{i_{q+1}},\cdots ,v_{i_{k}},v_{i_{q}},v_{i_{p}}}\\ \; & =0. \end{array}$$

This correction ensures that the hyper-adjacency matrix not only reflects the existence of a hyperedge but also incorporates the actual connectivity patterns within the hyperedge.

**Figure 3:**
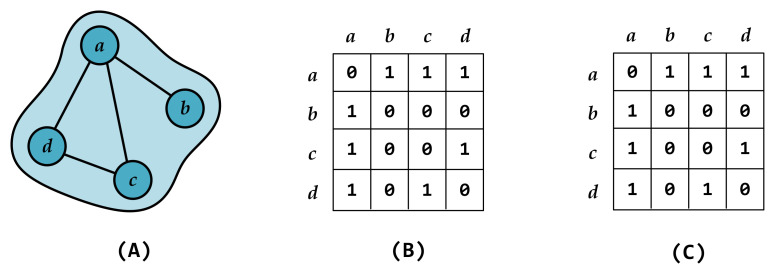
A schematic diagram of a four-node hyperedge with non-fully connected nodes. The hyperedge contains four nodes *a*, *b*, *c*, and *d*. In panel (**A**), the hyperedge shows that the edges {b,c} and {b,d} are absent, while all other pairs of nodes are connected. Panel (**B**) and panel (**C**) are matrices. The presence of an interaction between nodes is represented by 1, while 0 indicates that it does not exist. Specifically, panel (**B**) displays the observed connections among nodes within the hyperedge, and panel (**C**) shows the corrected hyper-adjacency matrix after dimensionality reduction.

Next, we take the four-node hyperedge from Figure 3 as an example to illustrate the corrected hyper-adjacency matrix. Figure 3A shows a hyperedge containing nodes *a*, *b*, *c*, and *d*, in which there are no edges between {b,c} and {b,d}, while all other pairs of nodes are connected. Table 1 presents the differences in elements between the traditional hyper-adjacency matrix and the corrected hyper-adjacency matrix in the higher-order representation. As shown in the table, in the corrected hyper-adjacency matrix, the higher-order interactions corresponding to combinations ending with {b,c}, {c,b}, {b,d}, or {d,b} no longer exist, reflecting the absence of edges between these node pairs. This is fundamentally different from the traditional hyper-adjacency matrix.

Finally, by performing dimensionality reduction on the hyper-adjacency matrix, a two-dimensional matrix can be obtained as follows:

(4) $$A_{ij}=\frac{1}{(k-2)!}\sum_{j_{2},\cdots ,j_{k-1}=1}^{N}{\left(A_{H_{k}}\right)}_{i\;j_{1}\cdots j_{k-1}}.$$

We introduce an additional normalization factor of (k−2)! for the high-order coupling function, ensuring that each interacting pair contributes equally to the system’s dynamics [31,32,33]. When considering the linearized dynamics around the fully synchronized state, such terms have been proven to be dynamically equivalent to those in Equation (1) [8]. In fact, as long as they are properly normalized by the aforementioned factor, their contribution to the Laplacian matrix remains consistent, as implemented in our formulation.

As illustrated in Figure 2, subfigure (c) shows the reduced two-dimensional matrices $A_{ij}^{\left(A\right)}$ and $A_{ij}^{\left(B\right)}$, obtained through dimensionality reduction from the corrected hyper-adjacency matrices shown in subfigure (b). These matrices describe the interactions between nodes that are connected within each hyperedge.

At the same time, we validate this approach using the four-node hyperedge in Figure 3 to ensure its effectiveness and consistency. Panel (B) shows the observed connections among nodes within the hyperedge, represented in an ordinary graph form, displaying only the pairwise interactions. Panel (C) shows the node-to-node associations obtained after dimensionality reduction of the corrected hyper-adjacency matrix according to Equation (4). We can see that the matrices in panel (B) and panel (C) are identical, indicating that our method is equivalent to the actual observed interactions. Therefore, in the following work, this newly proposed approach will be used to characterize the node-to-node associations within hyperedges.

In the corrected hyper-adjacency matrix, for each hyperedge, the connections among nodes are defined according to the actual hyperedge internal connectivity patterns rather than assuming complete connectivity by default. All interactions are assumed to be unweighted and undirected.

### 2.2. The Hypernetwork Synchronization Dynamics Model

For the synchronization dynamics in hypernetworks, we describe the evolution process under higher-order structures using the following equation:

(5) $${\dot{x}}_{i}=f\left(x_{i}\right)+\sum_{l=1}^{M}\zeta_{i}^{\left(l\right)}\;G^{\left(l\right)}\left({\left\{x_{j}\right\}}_{j\in e^{\left(l\right)}}\right),$$

(6) $$\zeta_{i}^{\left(l\right)}=\left\{\begin{array}{cc} 1, & i\in e^{\left(l\right)}\\ 0, & i\notin e^{\left(l\right)} \end{array}\right..$$

Each $G^{\left(l\right)}$ represents a multiple-interaction function, which typically depends on the states of all nodes within the hyperedge $e^{\left(l\right)}$. $\zeta_{i}^{\left(l\right)}$ is a selection function used to indicate whether the *l*-th hyperedge contains the *i*-th node.

Here, we adopt the hyper-adjacency matrix with corrected internal structures to describe the higher-order interactions within each hyperedge. For a hyperedge containing *k* nodes, its hyper-adjacency matrix is defined as follows:

(7) $$\begin{array}{cc} {\dot{x}}_{i} & =f\left(x_{i}\right)+\sum_{l=1}^{M}C^{\left(l\right)}\cdot A_{H_{k}}^{\left(l\right)}\left[\sum_{j\in e^{\left(l\right)}}H\left(x_{j}\right)-H\left(x_{i}\right)\right]\\ \; & =f\left(x_{i}\right)+\sum_{l=1}^{M}C^{\left(l\right)}\cdot \sum_{j_{2},\cdots ,j_{k-1}=1}^{N}a_{ij_{1}\cdots j_{k-1}}\left[\sum_{m=1}^{k-1}H\left(x_{j_{m}}\right)-H\left(x_{i}\right)\right]. \end{array}$$

Here, H(·) is the inner coupling function, representing the coupling term between nodes in the dynamical system.

By introducing the reduced matrix $A_{ij}^{\left(l\right)}$ derived from hyperedge *l* into Equation (7) and assuming that all hyperedges share the same coupling strength *C*, we obtain

(8) $${\dot{x}}_{i}=f\left(x_{i}\right)+C\sum_{l=1}^{M}\sum_{j\in e^{\left(l\right)}}A_{ij}^{\left(l\right)}\cdot \left[H\left(x_{j}\right)-H\left(x_{i}\right)\right],\;i=1,2,\cdots ,N$$

For the hyper-adjacency matrix with corrected internal structures for the hyperedges, we calculate the degree of each node within each hyperedge using the following method:

(9) $$d_{i}^{\left(l\right)}=\frac{1}{(k-1)!}\sum_{j_{1},j_{2},\cdots ,j_{k-1}=1}^{N}{\left(A_{H_{k}}\right)}_{i\;j_{1}\cdots j_{k-1}}.$$

Here, we normalize $A_{H_{k}}$ by a factor of (k−1)! to avoid repeated counting of the same higher-order structures [8].

### 2.3. The Laplacian Matrix and Its Stability

According to the adjacency matrix $A_{ij}^{\left(l\right)}$ of each hyperedge, this paper provides the Laplacian matrix $L_{ij}$ used to describe the hypernetwork. For $A_{ij}^{\left(l\right)}$, we use $L_{ij}^{\left(l\right)}$ as the Laplacian matrix of each hyperedge [8]:

(10) $$L_{ij}^{\left(l\right)}=d_{i}^{\left(l\right)}\;\delta_{ij}-A_{ij}^{\left(l\right)}$$

with the Kronecker delta $\delta_{ij}$. The Laplacian matrix used to describe the interactions of the nodes in each hyperedge is defined as

(11) $$L_{ij}=\sum_{l=1}^{M}L_{ij}^{\left(l\right)}.$$

Finally, we use $L_{ij}$ to describe the synchronization dynamics equation in non-uniform hypernetworks:

(12) $${\dot{x}}_{i}=f\left(x_{i}\right)+C\sum_{j=1}^{N}L_{ij}\;H\left(x_{j}\right),\;i=1,2,\cdots ,N.$$

In our work, we assume that the hypernetwork is undirected and unweighted, so the corresponding Laplacian matrix is symmetric and positive semi-definite. Therefore, all eigenvalues of the Laplacian matrix are non-negative and can be ordered as $0=\lambda_{1}<\lambda_{2}\leq \lambda_{3}\leq \cdots \leq \lambda_{\max}$ [34]. According to the master stability function method [22,35], when the node dynamics and inner coupling function are fixed, the synchronizability of the network can be assessed by checking whether all eigenvalues of its Laplacian matrix fall within the synchronization region. Specifically, for hypernetworks with an unbounded synchronization region, the synchronizability can be quantified by the smallest non-zero eigenvalue of the Laplacian matrix, $\lambda_{2}$. For hypernetworks with a bounded synchronization region, the synchronizability can be quantified by the eigenratio of the Laplacian matrix, $\lambda_{\max}/\lambda_{2}$ [36,37]. Since the measure of synchronizability is based on the master stability function, the synchronization considered in this paper refers to complete synchronization.

Compared with the undirected and unweighted case, a weighted hypernetwork has adjacency entries $A_{ij}^{\left(l\right)}$ representing interaction strengths rather than binary connections. The corresponding degrees become weighted degrees, and the Laplacian remains symmetric and positive semi-definite, but its spectrum is strongly affected by the distribution of the weights [38]. In contrast, a directed hypernetwork leads to asymmetric adjacency matrices, so the aggregated Laplacian is generally non-symmetric with possibly complex eigenvalues. In this case, the master stability function must be evaluated in the complex plane, and the synchronization manifold is determined by the left eigenvector associated with the zero eigenvalue [23,39]. Since this work mainly focuses on the general theoretical framework and numerical validation, we restrict our analysis to the undirected and unweighted case for clarity.

## 3. The Effect of Hyperedge Overlap on the Internal Structure

In this section, we will explore the effect of hyperedge overlap on the internal structure of the hypernetwork projection.

### 3.1. Probabilities in the Overlapping Region of the Hyperedges

To investigate the changes in substructures under hyperedge overlap, we use a projection graph instead of the original hypergraph. This is because the projection graph reduces the complexity of the hypernetwork while retaining the essential connections between nodes, making it easier to quantify the structural metrics. We characterize the hypernetwork using an equivalently reduced projection graph. Considering a hypernetwork with *M* hyperedges, each hyperedge corresponds to a reduced adjacency matrix, denoted as

(13) $$A_{ij}^{\left(l\right)},\;l=1,2,\cdots ,M.$$

The adjacency matrix of the projected graph, $A_{ij}^{\left(\mathrm{proj}\right)}$, is defined as

(14) $$A_{ij}^{\left(\mathrm{proj}\right)}=\left\{\begin{array}{cc} 1, & \mathrm{if}\;\sum_{l=1}^{M}A_{ij}^{\left(l\right)}\geq 1\\ 0, & \mathrm{otherwise} \end{array}\right..$$

In other words, the reduced matrices of all hyperedges are summed, and all non-zero elements are set to 1. This approach preserves all of the connection relationships between nodes while disregarding the accumulation of interaction strengths, as our focus here is on the structural changes between nodes.

In this study, we adopt the Erdős–Rényi (ER) model to describe the internal connections within hyperedges. The ER model is chosen for its well-defined and analytically tractable properties, such as the degree distribution and expected cycle counts, which facilitate the derivation of synchronization thresholds in closed form. Its inherent randomness also provides a simple and controllable baseline, allowing us to intuitively explore how the internal hyperedge connectivity influences network synchronization. In comparison, alternative models such as Barabási–Albert (BA) or Watts–Strogatz (WS) introduce additional complexity through power-law degree distributions or high clustering, which complicates the analytical treatment and reduces the generality.

**Figure 4:**
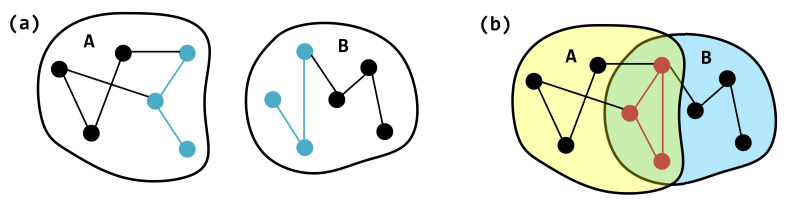
A schematic diagram of the new structure formed by the overlapping hyperedges. (**a**) Hyperedges A and B and their internal structure. (**b**) The new internal structure formed by the overlap of hyperedges A and B. The blue edges in (**a**) form a red cycle in (**b**). The green area is the overlapping area of hyperedge A and B, while the yellow and blue areas are the non-overlapping areas of A and B respectively.

In both hyperedges $e_{1}$ and $e_{2}$, the internal structure is modeled as an Erdős–Rényi (ER) graph with the connection probability *p*, denoted as G(p). Suppose these two hyperedges share *o* overlapping nodes, labeled $u_{1},u_{2},\cdots ,u_{o}$. In the projection graphs of hyperedges $e_{1}$ and $e_{2}$, we construct two ER subgraphs $G_{1}=G_{e_{1}}\left(p\right)$ and $G_{2}=G_{e_{2}}\left(p\right)$, respectively. *P* is the probability that there is an edge between *u* and *v*. Let the common node set be $V_{o}$; the induced subgraph on this node set is denoted as $G_{o}=G_{o}\left(p\right)$, which is also an ER graph. As shown in Figure 4, the projection graph of hyperedge *A* corresponds to $G_{1}$, and that of hyperedge *B* corresponds to $G_{2}$. The green region highlights the overlapping subgraph $G_{o}$ shared between the two projection graphs.

Within each hyperedge, the connections between node pairs are independent and occur with the probability *p*. Considering two common nodes $u,v\in e_{1}\cap e_{2}$, the edge events between them in the two independent graphs $G_{e_{1}}$ and $G_{e_{2}}$ are independent. We define $G_{o}$ as the union-induced subgraph on the common node set $V_{o}$ of the two hyperedges:

(15) $$G_{o}=G_{e_{1}}\left[V_{o}\right]\cup G_{e_{2}}\left[V_{o}\right],$$

where $G_{e_{1}}\left[V_{o}\right]$ and $G_{e_{2}}\left[V_{o}\right]$ denote the subgraphs induced by $V_{o}$ in $G_{e_{1}}$ and $G_{e_{2}}$, respectively.

Let event *A* denote that the edge (u,v) does not belong to the graph $G_{e_{1}}$, with the probability $P[\left(u,v\right)\notin G_{e_{1}}]=1-p$. Let event *B* denote that the edge (u,v) does not belong to the graph $G_{e_{2}}$, with probability $P[\left(u,v\right)\notin G_{e_{2}}]=1-p$. Since $G_{e_{1}}$ and $G_{e_{2}}$ are independently generated random graphs, events *A* and *B* are independent. Therefore, the probability that both events occur is

(16) $$P\left[\left(u,v\right)\notin G_{e_{1}}\;\mathrm{and}\;\left(u,v\right)\notin G_{e_{2}}\right]={\left(1-p\right)}^{2}.$$

Therefore, in $G_{o}$, the probability that the node pair (u,v) has an edge is equal to the probability that the edge exists in at least one of the two graphs, that is,

(17) $$P\left[\left(u,v\right)\in G_{o}\right]=1-P\left[\left(u,v\right)\notin G_{e_{1}}\;\mathrm{and}\;\left(u,v\right)\notin G_{e_{2}}\right]=1-{\left(1-p\right)}^{2}=2p-p^{2}.$$

This probability is evidently greater than *p* for p∈(0,1), and it increases monotonically as *p* increases.

Next, we calculate the expected number of cycles of length *l* in the projected graph, where *l* nodes are specified and arranged into a cycle. We define cycles as simple closed paths formed in the projected graph $G_{o}$, such as triangles, quadrilaterals, pentagons, and other cycles of length *l*.

Considering the construction of a cycle structure of length *l* on the common node set $V_{o}$ (where $|V_{o}|=o$),

- If o<l, it is impossible to form a closed path of length *l*, so the probability is zero;
- If o≥l, the number of ways to select *l* nodes from *o* and arrange them into a cycle is $\frac{o!}{(o-l)!}$, divided by 2l to avoid overcounting due to the starting point and direction.

In the projected graph $G_{o}$, the probability of an edge existing is $2p-p^{2}$, and a cycle of length *l* requires the existence of *l* specific edges. Therefore, the probability that each such cycle exists is ${\left(2p-p^{2}\right)}^{l}$.

Let $E[C_{l}]$ be the expected cycle count.Thus, the expected number of cycle structures of length *l* is given by

(18) $$E\left[C_{l}\right]=\left\{\begin{array}{cc} \frac{1}{2l}\cdot \frac{o!}{(o-l)!}\cdot {\left(2p-p^{2}\right)}^{l}, & o\geq l,\\ 0, & o<l. \end{array}\right.$$

The above expression clearly shows that when o≥l and p>0, $E[C_{l}]$ increases monotonically with the overlap size *o*. Since the edge existence probability $2p-p^{2}$ in the projected graph also increases with *p*, this indicates that the higher the hyperedge overlap, the greater the edge density in the projected graph, making it easier to form cycle structures within the common region.

### 3.2. Changes in Topological Properties in Projected Images

To further quantify the impact of hyperedge overlap on internal structural changes, this paper analyzes the structural properties of the hypernetwork’s projection graph using three indicators: the average degree, global clustering coefficient, and average closeness. Specifically, these three indicators are employed to characterize the substructures of the hyperedge overlap in the hypernetwork.

Consider a hypernetwork consisting of *M* hyperedges, where the number of nodes and the internal connection pattern within each hyperedge remain fixed. By adjusting the degree of overlap between hyperedges, we investigate how hyperedge overlap affects the overall structural properties.

#### 3.2.1. The Average Degree in Projected Images

First, the average degree of the graph is defined as

(19) $$d_{i}=\sum_{l=1}^{M}d_{i}^{\left(l\right)},\;\langle d\rangle =\frac{1}{N}\sum_{i=1}^{N}d_{i},$$

where $d_{i}$ denotes the degree of node *i*, and *N* is the total number of nodes.

In the experiment, to analyze the structural evolution of hypernetworks, we construct a hypernetwork consisting of two hyperedges, each containing 70 nodes. The internal connection patterns within the hyperedges are set as random (the ER model), scale-free (the BA model), and small-world (the WS model). We examine how different degrees of hyperedge overlap affect the structural properties and synchronization performance.

**Figure 5:**
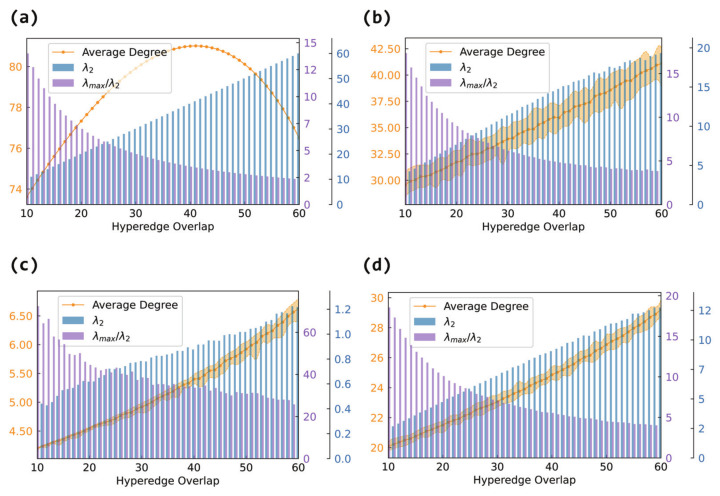
The effect of hyperedge overlap on hypernetwork synchronizability and the average degree of the projection graph. The horizontal axis represents hyperedge overlap, measured by the number of shared nodes between hyperedges. The left vertical axis corresponds to the line plot, which shows the average degree of the projection graph. The right vertical axis corresponds to the bar plot, where blue bars represent $\lambda_{2}$ and purple bars represent $\lambda_{\max}/\lambda_{2}$. Panel (**a**) shows the case of fully connected internal hyperedges, panel (**b**) shows the case of Erdős–Rényi (ER) internal connections, panel (**c**) shows the case of Barabási–Albert (BA) internal connections, and panel (**d**) shows the case of Watts–Strogatz (WS) internal connections.

In Figure 5, we track the average degree of the projection graphs as the number of overlapping nodes increases from 10 to 60. For non-fully connected internal structures (ER, BA, WS), the average degree shows a monotonic increase with the overlap in panels (b), (c), and (d). This aligns with our theoretical analysis: as the overlap between the hyperedges increases, more interconnections are formed between nodes, leading to denser projection graphs.

However, when each hyperedge is fully connected internally in panel (a), the average degree of the projection graph shows a non-monotonic trend: it first increases and then decreases. Initially, the overlap introduces many new links between the hyperedges, rapidly increasing the edge count. But as more nodes are shared, the edge redundancy increases, and the number of newly added effective connections declines, causing the average degree to drop.

#### 3.2.2. The Global Clustering Coefficient in Projected Images

Second, the global clustering coefficient *C* is used to measure the tendency of nodes to form triangular relationships, defined as

(20) $$C=\frac{3\times \mathrm{Number}\;\mathrm{of}\;\mathrm{triangles}}{\mathrm{Number}\;\mathrm{of}\;\mathrm{connected}\;\mathrm{triples}},$$

where the number of triangles refers to the number of closed triplets in the graph, and the number of connected triples indicates all paths of length two.

**Figure 6:**
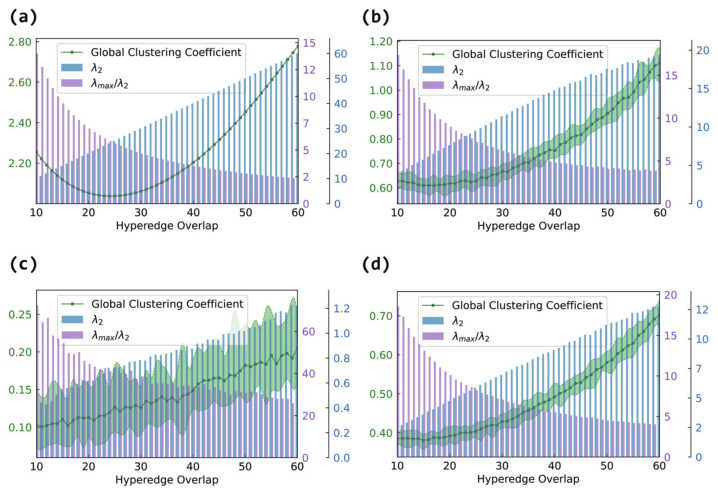
The effect of hyperedge overlap on hypernetwork synchronizability and the global clustering coefficient of the projection graph. The horizontal axis represents the hyperedge overlap, measured by the number of shared nodes between hyperedges. The left vertical axis corresponds to the line plot, which shows the average degree of the projection graph. The right vertical axis corresponds to the bar plot, where blue bars represent $\lambda_{2}$ and purple bars represent $\lambda_{\max}/\lambda_{2}$. Panel (**a**) shows the case of fully connected internal hyperedges, panel (**b**) shows the case of Erdős–Rényi (ER) internal connections, panel (**c**) shows the case of Barabási–Albert (BA) internal connections, and panel (**d**) shows the case of Watts–Strogatz (WS) internal connections.

Figure 6 presents the evolution of the global clustering coefficient under different overlap levels. For non-fully connected internal structures, the clustering coefficient steadily increases with the overlap in panels (b), (c), and (d). As more nodes are shared, the likelihood of triangle formation in the projection graph increases, boosting clustering.

In contrast, under fully connected internal structures in panel (a), the clustering coefficient displays a U-shaped trend: it first decreases and then increases. In the early stage of overlap, although many edges are added, they may not close triangles effectively, leading to a drop in clustering. As the overlap increases further, overlapping regions become densely interconnected (forming locally complete subgraphs), which sharply reduces the number of open triples and increases triangle formation, causing the clustering coefficient to rebound.

#### 3.2.3. Average Closeness in Projected Images

Finally, the average closeness is used to describe the average level of proximity among nodes. In a general graph G=(V,E), the closeness of node *i* is defined as

(21) $$C_{i}=\frac{N-1}{\sum_{i\neq j}d\left(i,j\right)},$$

where d(i,j) denotes the shortest path length between nodes *i* and *j*. The average closeness is defined as

(22) $$\langle C\rangle =\frac{1}{N}\sum_{i=1}^{N}C_{i}.$$

**Figure 7:**
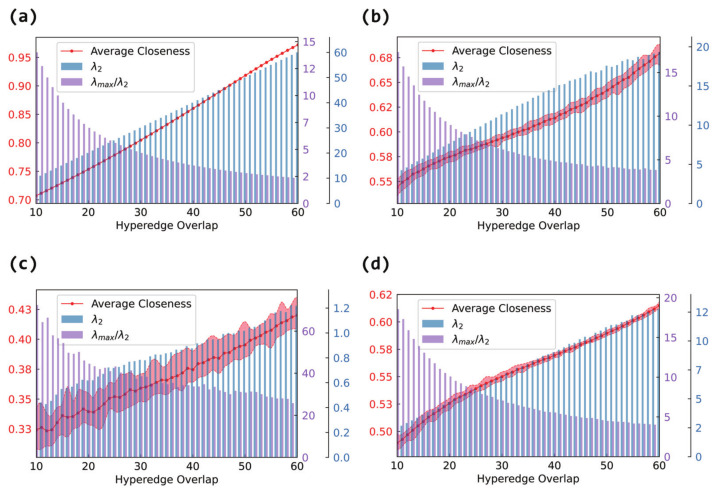
The effect of hyperedge overlap on hypernetwork synchronizability and the average closeness of the projection graph. The horizontal axis represents hyperedge overlap, measured by the number of shared nodes between hyperedges. The left vertical axis corresponds to the line plot, which shows the average degree of the projection graph. The right vertical axis corresponds to the bar plot, where blue bars represent $\lambda_{2}$ and purple bars represent $\lambda_{\max}/\lambda_{2}$. Panel (**a**) shows the case of fully connected internal hyperedges, panel (**b**) shows the case of Erdős–Rényi (ER) internal connections, panel (**c**) shows the case of Barabási–Albert (BA) internal connections, and panel (**d**) shows the case of Watts–Strogatz (WS) internal connections.

Figure 7 shows the variation in the average closeness in the projection graphs. Regardless of the internal structure, the average closeness increases consistently with the hyperedge overlap in panels (b), (c), and (d). More overlap leads to a denser network, reducing the shortest path lengths between node pairs and thereby improving the closeness.

This effect is especially pronounced when the internal structure is fully connected in panel (a). In this case, since the shortest paths are already minimized within each hyperedge, increasing the overlap further enhances inter-hyperedge connectivity, leading to a significantly larger rate of growth in the average closeness compared to that in non-fully connected cases. Even with the same number of overlapping nodes, the fully connected setting shows a much more substantial improvement in node accessibility.

In this work, we choose to quantify the structural properties using metrics from the projection graph rather than hypernetwork-specific parameters. The reason is that higher-order measures, such as the hyperclustering coefficient, cannot fully capture the effects of hyperedge overlap or the internal connectivity of hyperedges. For instance, overlapping hyperedges increase the node hyperdegrees and the network’s average hyperdegree, but this influence is not reflected in the hyperclustering coefficient, which only considers the relationships between hyperedges (e.g., the formation of hyper-triangles). The projection-based approach thus provides a practical and intuitive framework to quantify how overlapping and internally connected hyperedges shape the formation of substructures, laying the foundation for a subsequent analysis of the synchronization dynamics.

#### 3.2.4. Synchronizability in the Hypergraph

In this section, we will analyze the histograms in Figure 5 (Figure 6 and Figure 7). Since they are in the same network, the changes in $\lambda_{2}$ ($\lambda_{\max}/\lambda_{2}$) in each graph are the same. An increase in $\lambda_{2}$ and a decrease in $\lambda_{\max}/\lambda_{2}$ both indicate an enhancement in synchronizability, where the former applies to the case of unbounded synchronization regions and the latter to the case of bounded synchronization regions.

Although the internal connection patterns of the hyperedges differ, the experimental results consistently show that increasing the hyperedge overlap effectively enhances the global synchronizability of the hypernetwork. The magnitude of this improvement, however, depends on the specific internal structure. When the hyperedges are fully connected, $\lambda_{2}$ increases most rapidly (and $\lambda_{\max}/\lambda_{2}$ decreases most sharply), leading to the most pronounced synchronization enhancement. Random connections (Erdős–Rényi) follow, showing a substantial gain in connectivity. Small-world (Watts–Strogatz) connections exhibit an intermediate effect, lying between random and scale-free structures, while scale-free (Barabási–Albert) connections yield the smallest improvement.

Therefore, the internal connection pattern of the hyperedges has an important impact on the overall structural properties and synchronizability of hypernetworks. When considering internal hyperedge structures, increasing the hyperedge overlap improves the synchronizability to varying degrees, regardless of whether random, scale-free, small-world, or fully connected internal patterns are used. However, it is worth noting that different internal connection patterns lead to distinct trends in the structural evolution of the projection graphs, especially when the hyperedges are fully connected internally, where more complex non-monotonic characteristics are observed. Thus, it is meaningful to focus on the coupling effect of the internal hyperedge structures and hyperedge overlap when analyzing the synchronization mechanisms in hypernetworks.

## 4. Examples

In order to illustrate that the hyperedge overlap still has a significant impact on the synchronization ability of the hypernetwork in a hypernetwork that considers the internal structure of the hyperedges, we use the senate-bills dataset [40] and the senate-committees dataset [41] to verify the validity of the theory in the previous section.

The senate-bills dataset is a high-order hypernetwork composed of US senators, in which each hyperedge represents the sponsor and co-signer of a bill. The senate-committees dataset represents the relationship between a senator and their committee. These two datasets provide the modeling basis for real social high-order relationship networks from two different perspectives: the co-signing of bills and the composition of committee members. Therefore, they have the same set of nodes and different hyperedge associations between them. Both network construction methods have the property of a relatively stable hyperedge structure, but there is also overlap. In this analysis, the internal connections within each hyperedge are modeled using one of three standard random network types: Erdős–Rényi (ER), Barabási–Albert (BA), or Watts–Strogatz (WS). Since no empirical hypernetwork dataset captures the internal hyperedge structure, this approach provides a controllable framework for studying how different internal connectivity patterns affect global synchronization while remaining generalizable to real high-order networks.

As the interactive relationship between senators in co-signing bills or participating in committees evolves, their political positions, behavioral decisions, and opinions tend to be consistent. By numerically simulating the synchronization evolution process of these two actual hypernetworks, we demonstrate the impact of hyperedge internal structure modeling and the degree of overlap on the overall synchronizability, thereby verifying the applicability and explanatory power of the proposed theoretical framework in real high-order systems.

### 4.1. An Analysis of the Hypernetwork Structure

In this subsection, we will analyze the differences in the structures of the two empirical hypernetworks mentioned above. Specifically, we will analyze the node hyperdegree, the average degree of the hypernetwork projection graph, the global clustering coefficient, and the average closeness. We will also calculate the minimum non-zero eigenvalue of the Laplacian matrices of the two networks to characterize the difference in their synchronizabilities.

**Figure 8:**
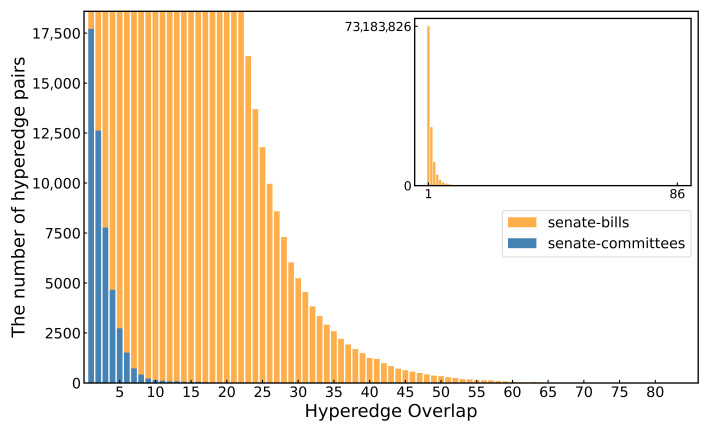
The hyperedge overlap distribution of the senate-bills and senate-committees hypernetworks. The horizontal axis represents the number of overlapping nodes between hyperedge pairs, while the vertical axis indicates the number of hyperedge pairs in the hypernetwork that exhibit each level of overlap.

Figure 8 illustrates the distribution of the hyperedge overlaps in the senate-bills and senate-committees hypernetworks. For each pair of hyperedges, we counted the number of overlapping nodes between them. From Figure 8, it can be observed that in the senate-committees hypernetwork, there are fewer hyperedge pairs with the same level of overlap. This indicates that the senate-committees hypernetwork exhibits relatively low hyperedge overlap. In contrast, the senate-bills hypernetwork generally shows a higher hyperedge overlap between hyperedges. Moreover, the average hyperedge overlap in the senate-committees hypernetwork is 1.9188928935308867, while in the senate-bills hypernetwork, it is 2.61470055554425. This suggests that on average, the hyperedges in the senate-bills hypernetwork share more common nodes, and thus the level of overlap between hyperedges is higher compared to that in the senate-committees hypernetwork.

Then, we compare the structural properties of the two hypernetworks with consideration of their hyperedge internal structures.

Table 2 presents a comparison of the projection graphs of the senate-bills and senate-committees hypernetworks in terms of the average degree, global clustering coefficient, and average closeness. The smaller $\lambda_{2}$ and the larger $\lambda_{\max}/\lambda_{2}$ of the Laplacian matrix is also included in the comparison. The larger values are highlighted in bold. From the table, we observe that the senate-bills hypernetwork exhibits higher values in all three structural parameters—the average degree, clustering coefficient, and average closeness—compared to those in the senate-committees hypernetwork. Additionally, its $\lambda_{2}$ is significantly larger, while its $\lambda_{\max}/\lambda_{2}$ is smaller. These results indicate that hypernetworks with higher structural metrics tend to possess stronger synchronizability, which is consistent with the theoretical conclusions derived in Section 3.

### 4.2. An Analysis of Hypernetwork Synchronization

In this subsection, we conduct numerical simulations to investigate the synchronization dynamics of empirical hypernetworks. The state evolution of each node is described by the classical Lorenz chaotic system [42], given as

(23) $$\left\{\begin{array}{c} \dot{x}=a\left(y-x\right),\\ \dot{y}=cx-xz-y,\\ \dot{z}=xy-bz, \end{array}\right.$$

where a,b,c are real-valued parameters. When a=10, b=8/3, and c=28, the system exhibits chaotic behavior. The coupling function between nodes is defined as H(·)=diag(1,0,0), and the coupling strength is set to 1. According to the master stability function (MSF) framework, the dynamical model in Equation (5) lies within the unbounded synchronization region. This conclusion holds under the above parameter settings. In this region, the MSF remains negative over an unbounded interval. This guarantees the potential for global synchronization. To quantify the degree of synchronization in the network [37], we define a global synchronization error function E(t) as follows:

(24) $$E\left(t\right)=\sum_{i=1}^{N}\sum_{j=1}^{N}\sum_{n=1}^{3}\sqrt{{\left({\hat{X}}_{in}\left(t\right)-{\hat{X}}_{jn}\left(t\right)\right)}^{2}},$$

where ${\hat{X}}_{in}\left(t\right)$ denotes the value of the *n*-th state variable of node *i* at time *t*. If $E\left(t\right)<10^{-6}$ at a given time *t*, the network is considered to have achieved global synchronization; otherwise, it is deemed unsynchronized. The numerical integration of the differential equations in (23) is performed using the classical fourth-order Runge–Kutta method, ensuring both accuracy and numerical stability in the simulation process.

**Figure 9:**
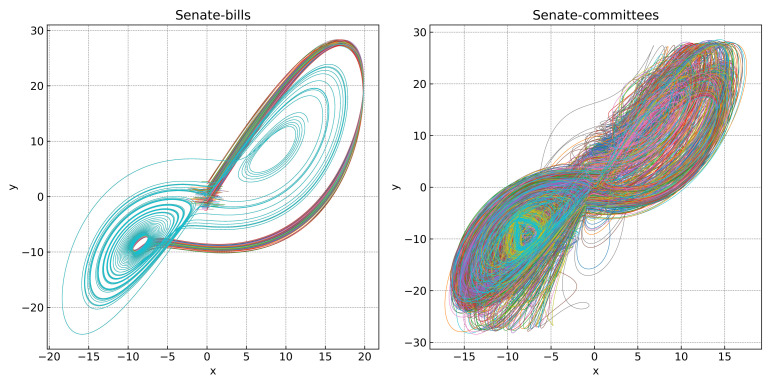
The state evolution of the nodes in the senate-bills and senate-committees hypernetworks. Each line in the figure is represented by a different color, representing the state change of a node in the hypernetwork.

Figure 9 shows the state evolution of the nodes in the Lorenz system, where the trajectory is projected onto the *z* plane by visualizing the relationship between the *x* and *y* components. As illustrated in the figure, for the senate-bills hypernetwork, the node states rapidly converge and overlap into a single trajectory (blue curve), indicating synchronization. In contrast, in the senate-committees hypernetwork, the node states fail to fully overlap, and synchronization is not achieved. This demonstrates that under an identical coupling strength (C=1), the senate-bills hypernetwork reaches a synchronized state over time, where the senate-committees hypernetwork does not.

**Figure 10:**
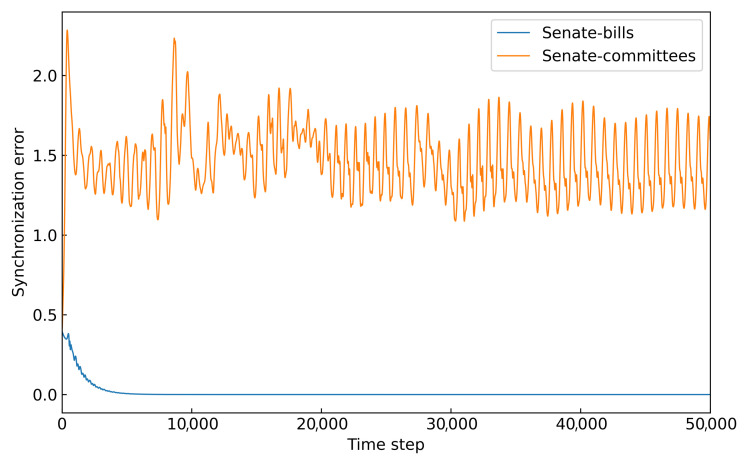
The synchronization error of the senate-bills and senate-committees hypernetworks. The horizontal axis represents the time step, and the vertical axis represents the error between all node states. It is calculated using Equation (24).

Furthermore, we compute the state error between nodes in the two hypernetworks, as shown in Figure 10. It can be observed that under the same coupling strength (C=1), the synchronization error in the senate-bills hypernetwork rapidly approaches zero as time progresses, indicating successful synchronization. However, in the senate-committees hypernetwork, the node error remains large throughout, and synchronization fails to occur.

Through numerical simulations, we conclude that the senate-bills hypernetwork, characterized by richer substructures, exhibits a significantly better synchronization capability compared to that of the senate-committees hypernetwork.

## 5. Conclusions

In this study, we explored the impact of hyperedge overlap on the global synchronizability of hypernetworks, taking into full account the internal structure of the hyperedges. The results show that as the hyperedge overlap increases, the probability of existing connections between nodes in the hypernetwork also increases, thereby enhancing the synchronizability of the hypernetwork. Specifically, using the average degree, global clustering coefficient, and average closeness of the projection graph as evaluation metrics, we observed that increasing the hyperedge overlap significantly improves these structural parameters in hypernetworks with scale-free, random, and small-world internal connection patterns. Meanwhile, the global synchronizability of the hypernetwork also improves, indicating that even when considering the internal connections among nodes within hyperedges, hyperedge overlap remains a key factor influencing hypernetwork synchronizability. Furthermore, by combining empirical networks with an eigenvalue analysis and numerical simulations, we validated the universality of the above conclusions in large-scale hypernetworks. The results show that hypernetworks with a higher overlap of hyperedges consistently exhibit a better synchronization performance than those with a lower overlap, even when the internal structure of the hyperedges is explicitly defined. This confirms that the effect of hyperedge overlap on synchronizability cannot be ignored. This provides theoretical support for the study of hypernetwork synchronizabilities considering the hyperedges’ internal structure.

In this study, we use the projection graph approach to characterize the internal structure of the hyperedges in hypernetworks. This method, however, has certain limitations, as it cannot fully capture both hyperedge interactions and internal connectivity simultaneously. Future work should aim to develop structural metrics that reflect not only the relationships between hyperedges but also the internal organization within each hyperedge. Moreover, future research will focus on systematically exploring the features of the internal hyperedge structure, particularly identifying which internal connectivity patterns are most conducive to enhancing global synchronization under specific hyperedge interactions. By constructing more targeted internal structure models, we aim to provide clearer and more effective theoretical guidance for optimizing synchronization in real-world hypernetwork systems. These insights could inform the design and control of complex systems where high-order interactions play a crucial role, such as collaborative decision-making networks, multi-agent coordination systems, and social or biological networks, enabling more robust and efficient collective dynamics.

## Figures and Tables

**Table 1:** Comparison between traditional and corrected hyper-adjacency matrices.

Elements	Traditional	Corrected	Elements	Traditional	Corrected
[a, b, c, d]	1	1	[b, a, c, d]	1	1
[a, b, d, c]	1	1	[b, a, d, c]	1	1
[a, c, b, d]	1	0	[b, c, a, d]	1	1
[a, c, d, b]	1	0	[b, c, d, a]	1	1
[a, d, b, c]	1	0	[b, d, a, c]	1	1
[a, d, c, b]	1	0	[b, d, c, a]	1	1
[c, a, b, d]	1	0	[d, a, b, c]	1	0
[c, a, d, b]	1	0	[d, a, c, b]	1	0
[c, b, a, d]	1	1	[d, b, a, c]	1	1
[c, b, d, a]	1	1	[d, b, c, a]	1	1
[c, d, a, b]	1	1	[d, c, a, b]	1	1
[c, d, b, a]	1	1	[d, c, b, a]	1	1

**Table 2:** This table is used to compare the properties of two hypernetworks.

	Senate-Bills	Senate-Committees
Average Degree	**135.55**	74.66
Clustering Coefficient	**1.8163**	0.9268
Average Closeness	**0.7717**	0.6195
$\lambda_{2}$	**1219.9016**	30.9569
$\lambda_{\max}/\lambda_{2}$	15.5565	**18.6483**

## Data Availability

The data and codes used for this study are available at https://github.com/155641/Effects-of-Hyperedge-Overlap-and-Internal-Structure-on-Hypernetwork-Synchronization-Dynamics.git (accessed on 21 July 2025).
